# Fetal behavior as a window to later gross motor function: a 10-year follow-up of children prenatally exposed to gestational diabetes mellitus

**DOI:** 10.3389/fcdhc.2026.1938676

**Published:** 2026-09-11

**Authors:** Oliver Vasilj, Dora Marinić, Tatjana Trošt, Zoran Žeželj

**Affiliations:** 1 Department of Nursing, University North, Varaždin, Croatia; 2 Faculty of Kinesiology, University of Zagreb, Zagreb, Croatia

**Keywords:** fetal behavior, follow-up study, gestational diabetes mellitus, gross motor development, neuromotor development

## Abstract

**Introduction:**

Gestational diabetes mellitus (GDM) has been associated with adverse neurodevelopmental outcomes in early childhood, but evidence regarding its long-term association with gross motor functioning remains limited. This 10-year follow-up study examined gross motor outcomes at approximately ten years of age in children prenatally exposed to diet-treated GDM and children from uncomplicated pregnancies, building on a prospectively established cohort in which fetal neurobehavior had previously been assessed.

**Methods:**

The study included 100 children, with 50 participants in each group. Gross motor functioning was assessed across six domains: coordination, agility, flexibility, explosive strength, muscular endurance, and balance. Given that several motor outcomes deviated from normality, Aligned Rank Transform (ART) factorial ANOVA was used to examine the effects of group, sex, and the group × sex interaction. Benjamini–Hochberg false discovery rate correction was applied to the p-values for the main effect of group to account for multiple comparisons.

**Results:**

Gross motor functioning was largely comparable between the groups, with no significant main effect of group for most motor outcomes or any balance parameter. However, significant group effects were identified for three specific motor tasks and remained statistically significant after FDR correction: children in the GDM-exposed group performed poorer in squats and ball rolling with the non-dominant hand, while they performed better in the straddle forward bend. The magnitude of the group effects was moderate for squats and straddle forward bend and large for ball rolling with the non-dominant hand.

**Discussion:**

These findings do not indicate generalized gross motor impairment but suggest that prenatal exposure to the metabolic environment associated with GDM may be related to subtle, task-specific differences in motor functioning that remain detectable during school age. Further longitudinal research is needed to determine the developmental stability and clinical relevance of these outcomes.

## 1 Introduction

Gestational diabetes mellitus (GDM) is defined as diabetes first diagnosed or recognized during pregnancy, and its effects may influence the course of pregnancy, childbirth, and later development (1–4). Increasing attention is being given to its possible long-term effects on health, including metabolic and neurodevelopmental outcomes (3–5). Maternal hyperglycemia may affect fetal development through mechanisms consistent with the Developmental Origins of Health and Disease (DOHaD) concept. According to this concept, adverse conditions during sensitive periods of development may lead to long-lasting structural and functional adaptations that affect health throughout life (6). In pregnancies complicated by GDM, the fetus is exposed to an altered metabolic environment that may influence the maturation and functioning of different organ systems, including the central nervous system. These effects do not necessarily have to be visible at birth or during infancy. They may become apparent later in childhood, when neurodevelopmental demands become more complex (2, 3). Recent studies have also reported structural differences in various areas of the brain associated with the altered intrauterine metabolic environment caused by gestational and pregestational diabetes (7–11). Ahmed et al. (7) conducted a study examining the effects of maternal diabetes on brain development and neurocognitive functioning in preadolescent children. Their findings showed reduced cortical thickness in most areas of the occipital lobe in both hemispheres, as well as in the right postcentral gyrus and the left superior parietal cortex. These changes may increase the risk of atypical brain development, including processes such as inadequate axonal myelination during childhood. Van Dam et al. (10) found that gestational diabetes may cause dysfunction of the central nervous system through reduced neural excitability and plasticity. The literature also reports microstructural abnormalities of white matter in newborns from pregnancies complicated by gestational diabetes (11), reduced volume of certain hippocampal subregions, and sex-specific changes in hippocampal morphology during childhood among children exposed to GDM *in utero* (8). All of these changes may be associated with difficulties in psychomotor functioning during childhood, adolescence, and adulthood. These findings provide a neurophysiological basis for the assumption that prenatal exposure to GDM may be linked to long-term functional differences in childhood. However, the functional significance of these findings is still not fully understood. Gross motor abilities are an important neurodevelopmental indicator because successful motor performance requires the coordinated interaction of motor planning and execution, sensory feedback, visuomotor processing, and postural control (12–14). Motor and cognitive development in children are closely connected, especially when children perform complex motor tasks that cannot be completed without typical cognitive functioning (15, 16). For this reason, assessing gross motor abilities may help determine whether an altered metabolic environment during fetal life is associated with long-term developmental outcomes (3, 7–11). In this context, tasks involving coordination, balance, and visuomotor integration may provide valuable information because they require the integration of several sensorimotor and cognitive processes (13, 15, 16). Previous studies have found a negative association between maternal GDM and later motor development in offspring (3, 5, 17). Differences in spontaneous movements have already been observed during fetal life, which is the earliest period in which neuromotor functioning can be assessed in a standardized way (20). During infancy and early childhood, children born from pregnancies complicated by GDM showed poorer results in different areas of motor development (21–27). Some studies suggest that developmental differences become less noticeable with age, while others report differences in fine and gross motor skills or neuropsychological functioning during school age and adolescence as well (18, 19, 28). Although previous studies clearly point to the long-term effects of gestational diabetes on gross motor development, most have focused on infancy, early childhood, or preschool age. In addition, the contradictions mentioned above mainly come from cross-sectional studies. Most studies present gross motor development through a total score and include only a small number of tested variables. As a result, it is still not entirely clear whether prenatal exposure to the altered metabolic environment associated with GDM leads to poorer gross motor development later in childhood, or whether these abilities eventually become comparable to those of peers born from healthy pregnancies. Building on the previously conducted standardized assessment of fetal behavior in pregnancies complicated by GDM (20), the aim of this 10-year follow-up study was to determine whether prenatal exposure to gestational diabetes is associated with differences in specific gross motor outcomes at approximately ten years of age.

## 2 Materials and methods

### 2.1 Study design

This study represents a 10-year follow-up of a prospectively established cohort. Participants were initially assessed during the fetal period and were re-evaluated at approximately ten years of age. The initial assessment was conducted during the third trimester of pregnancy, between 32 and 38 weeks of gestation, when fetal neurobehavior was assessed; the results of this assessment have been previously published. The present follow-up assessment was conducted approximately ten years later and focused on gross motor functioning during school age.

### 2.2 Participants

The initial sample (20) included pregnant women with singleton pregnancies between 32 and 38 weeks of gestation. The GDM-exposed group consisted of 82 pregnant women diagnosed with gestational diabetes mellitus (GDM), while the unexposed group consisted of 82 pregnant women with uncomplicated pregnancies and normal oral glucose tolerance test (OGTT) results. GDM was diagnosed using a 75-g OGTT according to the HAPO criteria (29): fasting plasma glucose ≥5.1 mmol/L, 1-hour plasma glucose ≥10.0 mmol/L, and 2-hour plasma glucose ≥8.5 mmol/L. All women with GDM were managed with dietary treatment only, and none received insulin therapy. Non-inclusion criteria at the initial fetal assessment included pathological maternal or fetal conditions that could potentially affect fetal behavior, including intrauterine growth restriction, pathological Doppler findings, abnormal cardiotocography or biophysical profile findings, structural or chromosomal fetal abnormalities, hydrops fetalis, preeclamptic disorders, hemolytic disease, thrombophilia, threatened preterm labor, intra-amniotic infection syndrome, viral and other infections including TORCH infections and parvovirus B19, severe psychiatric disorders, abuse of medications, narcotics or alcohol, and smoking.

A follow-up assessment during childhood was planned from the outset of the cohort, although the exact age of reassessment was not predefined. The present follow-up was conducted at approximately ten years of age, prior to the period in which pubertal maturation typically introduces substantial biological and psychomotor changes that may influence motor performance and complicate the interpretation of gross motor outcomes.

At the 10-year follow-up, 55 of the original 82 participants in the GDM-exposed group and 54 of the original 82 participants in the unexposed group were successfully contacted, corresponding to contact rates of 67.1% and 65.9%, respectively. The remaining families could not be reached because their contact information was no longer valid due to changes in telephone numbers and/or addresses. Children were excluded from the 10-year follow-up assessment if they had developmental difficulties associated with genetic factors, perinatal or postnatal complications, or acquired impairments. In the GDM-exposed group, two children were excluded, one due to Down syndrome and one due to cerebral palsy, two families had moved abroad, and one family subsequently declined participation, resulting in a final sample of 50 children. In the unexposed group, three families did not participate because of family- or health-related reasons, and one child was excluded after developmental difficulties were identified during the follow-up assessment, resulting in a final sample of 50 children. The GDM-exposed group included 36 girls and 14 boys, whereas the unexposed group included 20 girls and 30 boys. The mean age of the children was 10 years, with an age range from 9 years and 3 months to 11 years and 1 month. At the 10-year follow-up, participants in the GDM-exposed and unexposed groups did not differ in anthropometric characteristics, including body weight, height, and body fat percentage, as shown in **Table 1**.

**Table 1:** Anthropometric characteristics of participants in the unexposed and GDM-exposed group.

Variable	Unexposed group	GDM-exposed group	p-value
Height	145.88 ± 6.96	147.64 ± 6.27	0.36
Body weight	36.5 ± 9.73	39.35 ± 11.25	0.29
Body fat percentage	21.88 ± 6.49	23.91 ± 6.82	0.20

Values are presented as mean ± standard deviation. Between-group differences were tested using the independent-samples t-test or Mann–Whitney U test, as appropriate. GDM, gestational diabetes mellitus.

### 2.3 Assessment of fetal behavior

Fetal behavior was assessed during the initial measurement point of the longitudinal study. The assessment was performed during the third trimester of pregnancy, between 32 and 38 weeks of ultrasonographically confirmed gestation. Fetal neurobehavior was evaluated using the Kurjak Antenatal Neurodevelopmental Test (KANET), in its modified version according to Mišković et al. (30). Fetal neurobehavioral assessments were performed by a specialist in obstetrics and gynecology with subspecialty training in fetal medicine, using the standardized modified KANET protocol. The assessor was blinded to the participants’ GDM exposure status at the time of fetal neurobehavioral assessment. The assessment was conducted using four-dimensional ultrasound (4D ultrasound), which enables the evaluation of both the quantity and quality of fetal movements.

The modified KANET protocol includes the assessment and scoring of seven groups of fetal signs and movements: isolated head anteflexion, isolated eye blinking and facial expressions, mouth movements, isolated leg movements, isolated hand movements and hand-to-face movements, finger movements, and the overall impression of general movements. Each item is scored from 1 to 3, with higher scores indicating more optimal fetal neurobehavior. The results of the fetal behavior assessment used as the initial assessment of the present cohort have been previously published by Vasilj et al. (20).

### 2.4 Assessment of gross motor development

Gross motor development was assessed during the second measurement point, when the children were ten years old. The assessment included six domains of motor abilities: coordination, agility, flexibility, explosive strength, muscular endurance, and balance. Coordination, agility, flexibility, explosive strength, and muscular endurance were assessed using tests from the CROFIT norm battery (31), whereas balance was assessed separately using a quiet standing with eyes closed test on a specially designed unstable platform with the Gyko system (32). Gross motor assessments were conducted by a Master of Kinesiology in the Laboratory for Motor Research, following standardized testing procedures. The assessor was blinded to the participants’ prenatal GDM exposure status.

#### 2.4.1 Coordination

Coordination was assessed using the Backwards Obstacle Course test and the Ball Rolling with the Non-Dominant Hand test. In both tests, performance time was recorded in hundredths of a second, with shorter time indicating better performance. In the Backwards Obstacle Course test, the participant moved backward on all fours along a 10-meter course, passing over one obstacle and under another obstacle, both 50 cm in height. In the Ball Rolling with the Non-Dominant Hand test, the participant rolled a ball with the non-dominant hand between and around stands placed along a 4-meter course. Both tests were performed three times, and the arithmetic mean of the results was used for analysis.

#### 2.4.2 Agility

Agility was assessed using the Side Steps test and the Shuttle Run Transfer test. In both tests, performance time was recorded in hundredths of a second, with shorter time indicating better performance. In the Side Steps test, the participant moved laterally using side steps between two lines placed 4 meters apart, completing the movement six times. In the Shuttle Run Transfer test, the participant ran a distance of 9 meters to transfer two sponges, one at a time, back to the starting line. Both tests were performed three times.

#### 2.4.3 Flexibility

Flexibility was assessed using the Straddle Forward Bend test and the Bench Forward Bend test. Reach distance was recorded in centimeters, with higher values indicating better performance. In the Straddle Forward Bend test, the participant sat with the back against a wall and the legs spread apart, and then performed a maximal forward bend by reaching forward with both hands along the floor. In the Bench Forward Bend test, the participant stood with both feet on a 40-cm-high bench and performed a maximal forward bend without bending the knees.

#### 2.4.4 Explosive strength

Explosive strength was assessed using the Standing Long Jump test and the 20-meter Sprint from a Standing Start test. In the Standing Long Jump test, jump distance was recorded in centimeters, with greater distance indicating better performance. In the 20-meter Sprint test, performance time was recorded in hundredths of a second, with shorter time indicating better performance. In the Standing Long Jump test, the participant performed a two-footed forward jump from a take-off board, attempting to jump as far as possible. In the 20-meter Sprint from a Standing Start test, the participant ran 20 meters at maximal speed from a standing start.

#### 2.4.5 Muscular endurance

Muscular endurance was assessed using the Sit-Up test and the Squat test. In both tests, the number of correctly performed repetitions within one minute was recorded, with a higher number of repetitions indicating better performance. In the Sit-Up test, the participant started from a supine position with the legs flexed at a 90-degree angle and performed as many sit-ups as possible within one minute. In the Squat test, the participant performed consecutive squats from a standing position until touching the floor with the fingers, also within one minute.

#### 2.4.6 Balance

Balance was assessed using the Quiet Standing with Eyes Closed on an Unstable Surface test. The participant stood in a slightly open stance on a platform that rotated in the anterior-posterior direction. The task was performed three times, with each trial lasting 30 seconds. Using the Gyko system, the following platform displacement variables were recorded: path length (LEN), velocity of displacement (VEL), mean distance (MD), mean frequency (MF), and root mean square distance (RMS).

### 2.5 Statistical analysis

All variables were first analyzed using descriptive statistics, including minimum and maximum values and arithmetic means. Normality of the distributions was assessed separately for the GDM-exposed and unexposed groups using the Shapiro–Wilk test. Between-group differences in anthropometric characteristics were examined using the independent-samples t-test when the data in both groups were normally distributed and the Mann–Whitney U test when the distribution in at least one group deviated from normality.

For motor outcomes, given that several variables deviated from normality and the subgroup sizes were relatively small, an Aligned Rank Transform (ART) factorial ANOVA was used to examine the effects of prenatal GDM exposure and sex. The model included group (GDM-exposed vs. unexposed), sex, and the group × sex interaction. Effect sizes were expressed as partial eta squared (ηp²), with values of approximately 0.01, 0.06, and 0.14 interpreted as small, moderate, and large effects, respectively.

To account for multiple comparisons, Benjamini–Hochberg false discovery rate (FDR) correction was applied to p-values for the main effect of group. The correction was performed separately for the motor performance outcomes and the balance parameters. The five balance parameters were derived from the same quiet-standing assessment and were therefore treated as a separate family of related balance outcomes. Sex was included in the factorial model to account for its potential contribution to motor performance; however, sex-related differences were not a primary hypothesis of the present study, and FDR correction was therefore not applied to the p-values for the main effect of sex.

A *post hoc* power analysis conducted using G*Power showed that the final sample of 100 participants provided a statistical power of 0.74 for detecting a medium effect size. Given that this value was below the conventional threshold of 0.80, non-significant findings were interpreted cautiously. The level of statistical significance was set at p < 0.05. Descriptive statistics and between-group comparisons of anthropometric characteristics were conducted using IBM SPSS Statistics (version 25.0), while ART analyses were performed in R (version 4.5.2) using the ARTool package.

## 3 Results

Descriptive statistics and the results of the factorial ART analyses for gross motor outcomes are presented in **Table 2**. Significant main effects of group were observed for the squat test, ball rolling with the non-dominant hand, and the straddle forward bend. All three group effects remained statistically significant after Benjamini–Hochberg FDR correction. Children in the GDM-exposed group performed poorer in the squat test (FDR-adjusted p = 0.027, ηp² = 0.079) and ball rolling with the non-dominant hand (FDR-adjusted p = 0.001, ηp² = 0.152), whereas they performed better in the straddle forward bend (FDR-adjusted p = 0.027, ηp² = 0.072). The effect sizes were moderate for the squat test and straddle forward bend and large for ball rolling with the non-dominant hand.

**Table 2:** Descriptive statistics and between-group differences in gross motor skills between the unexposed and GDM-exposed group.

Variable	Unexposed group	GDM group	Group p	Group p FDR	Group ηp²	Sex p	Sex ηp²	Interaction p	Interaction ηp²
Sprint	5.05 ± 0.32	5.24 ± 0.70	0.31	0.52	0.01	< 0.01	0.09	< 0.01	0.09
Standing long jump	136.69 ± 11.26	129.85 ± 24.38	0.5	0.55	0.01	< 0.01	0.10	0.10	0.03
Lateral steps	12.75 ± 1.32	13.20 ± 1.74	0.44	0.55	0.01	< 0.01	0.19	0.37	0.01
Shuttle run	13.30 ± 1.11	13.66 ± 1.49	0.37	0.52	0.01	< 0.01	0.11	0.01	0.06
Sit-ups	31.38 ± 7.00	25.64 ± 11.56	0.06	0.16	0.04	0.02	0.06	0.18	0.02
**Squats**	**40.88 ± 5.42**	**36.04 ± 9.36**	**0.01**	0.03	**0.08**	**0.01**	**0.07**	**0.07**	**0.03**
Backward obstacle course	19.43 ± 4.49	21.56 ± 8.04	0.69	0.69	< 0.01	0.18	0.02	0.14	0.02
**Ball rolling with the non-dominant hand**	**24.09 ± 4.20**	**29.07 ± 8.53**	**< 0.01**	**< 0.01**	**0.15**	**< 0.01**	**0.32**	**0.05**	**0.04**
**Straddle forward bend**	**50.19 ± 9.93**	**55.71 ± 14.30**	**0.01**	0.03	**0.07**	**< 0.01**	**0.27**	**0.08**	**0.03**
Bench forward bend	−4.40 ± 7.81	−1.43 ± 8.08	0.10	0.20	0.03	< 0.01	0.17	0.82	< 0.01
Length of platform displacement	16.19 ± 9.21	17.97 ± 13.32	0.99	0.99	< 0.01	0.88	< 0.01	0.05	0.04
Mean distance of platform displacement	0.17 ± 0.10	0.19 ± 0.14	0.50	0.99	0.01	0.71	< 0.01	0.16	0.02
Root mean square of platform displacement	0.20 ± 0.13	0.24 ± 0.18	0.40	0.99	0.01	0.70	< 0.01	0.14	0.02
Velocity of platform displacement	0.54 ± 0.31	0.60 ± 0.44	0.93	0.99	< 0.01	0.99	< 0.01	0.03	0.05
Mean frequency of platform displacement	0.75 ± 0.31	0.77 ± 0.30	0.46	0.99	0.01	0.77	< 0.01	0.01	0.08

Values are presented as mean ± standard deviation. Main effects of group and sex and group × sex interactions were examined using Aligned Rank Transform (ART) factorial ANOVA. Effect sizes are reported as partial eta squared (ηp²). Benjamini–Hochberg false discovery rate (FDR) correction was applied to p-values for the main effect of group, separately for motor performance outcomes and balance parameters. GDM, gestational diabetes mellitus; FDR, false discovery rate.

Bold values indicate statistically significant group effects that remained significant after FDR correction (p < 0.05).

No significant main effects of group were observed for sprint, standing long jump, lateral steps, shuttle run, sit-ups, backward obstacle course, bench forward bend, or any of the balance parameters. Sex showed significant main effects for several motor performance outcomes. Significant group × sex interactions were observed for sprint, shuttle run, and ball rolling with the non-dominant hand. For the balance assessment, no significant main effects of group were observed; however, significant group × sex interactions were found for path length, velocity, and mean frequency.

## 4 Discussion

The main finding of this study is that children born from pregnancies complicated by GDM, at approximately ten years of age, did not differ from children in unexposed group in most of the assessed gross motor abilities. No significant differences were found in agility, explosive strength, balance, or in most measures of coordination, muscular endurance, and flexibility. Differences were found in three specific tasks: children in the GDM group achieved poorer results in squats and rolling a ball with the nondominant hand, while they performed better in the straddle forward bend. This pattern of results does not suggest a general impairment in gross motor abilities, but rather possible specific differences in individual tasks that place different motor, sensory, and cognitive demands on the child. Importantly, these three group differences remained statistically significant after accounting for sex in the factorial ART analysis and after Benjamini–Hochberg false discovery rate correction for multiple comparisons. The magnitude of the group effects was moderate for squats and straddle forward bend and large for ball rolling with the nondominant hand. These findings indicate that the observed group differences persisted after accounting for sex and correction for multiple comparisons. At the same time, the significant contribution of sex to several motor outcomes highlights the importance of considering sex when interpreting motor performance in this age group.

This finding is partly consistent with the results of Kolseth et al., who did not find clear differences in physical health and neurodevelopmental outcomes among seven-year-old children whose mothers participated in an intervention study during pregnancy because they were at increased risk of developing GDM (38). Still, comparison with that study should be made with caution because its population did not include only children of mothers with confirmed GDM. On the other hand, the meta-analysis by Arabiat et al. showed that exposure to gestational diabetes during fetal life was associated with poorer motor development, including gross motor development (17). However, the authors pointed out considerable variability in the results and the possible contribution of diabetes type, maternal obesity, hypertension, socioeconomic factors, and other pregnancy-related characteristics (17). Differences have also been found in some studies involving school-aged children. Bolaños et al. found poorer graphic, spatial, and bimanual abilities, a higher number of signs of minor neurological dysfunction, and differences in certain cognitive measures among school-aged children born from pregnancies complicated by GDM (18). Long-term outcomes may depend on the child’s developmental age at the time of assessment, the severity of gestational diabetes, the level of glycemic control, associated metabolic conditions, and the sensitivity of the assessment tools used (3, 5, 26). In addition, significant group × sex interactions observed for selected motor outcomes suggest that the association between prenatal GDM exposure and motor performance may not be uniform across boys and girls. These interaction effects should be interpreted cautiously and warrant further investigation in larger, sex-balanced samples.

In this study, no difference was found in coordination during the backward obstacle course, while children in the GDM group achieved a poorer result when rolling a ball with the nondominant hand. Different results in two tests within the same broader motor domain may suggest that the observed difference does not reflect generally poorer coordination, but rather the specific demands of the task. Rolling a ball with the nondominant hand requires coordination of the upper limbs, continuous adjustment of movement direction and intensity, spatial orientation, visual tracking, and control of the nondominant side of the body (31). The poorer result may therefore be related to the greater demands placed on visuomotor integration and bimanual organization of movement. This interpretation is partly consistent with the findings of Bolaños et al., who reported poorer graphic, spatial, and bimanual abilities and a higher frequency of minor neurological signs among school-aged children born from pregnancies complicated by GDM (18). The relatively large group effect observed for this task suggests that between-group differences may be more apparent in motor tasks involving greater visuomotor and coordination demands.

In muscular endurance, no difference was found in the sit-ups test, while children in the GDM group completed a significantly lower number of squats within the given time. Again, this pattern does not support the conclusion that their overall muscular endurance was poorer. Instead, differences between the tasks themselves should be considered. The squat test involves repeated dynamic activation of the lower-limb and trunk muscles, control of body position, maintenance of balance, and effective temporal and spatial organization of a repeated movement pattern (31). Since similar squat tests were generally not used in earlier studies, this result is difficult to compare with the existing literature (17). The poorer performance may reflect differences in the dynamic endurance of the lower limbs, the efficiency of repeated movement, postural control, or coordination between these components.

The better result of the GDM group in the straddle forward bend was unexpected, particularly because no significant difference between the groups was found in the other flexibility test. Studies comparing different versions of forward bend tests have shown that the results of individual protocols are not fully interchangeable and that the tests may differ in how closely they are related to hamstring flexibility (33). The straddle forward bend and the bench forward bend test are performed from different starting positions. They also differ in the position of the lower limbs, pelvis, and trunk, which means that they may not reflect the same components of flexibility equally (31, 33).

One potential biological factor, although still insufficiently studied, may be the hormone relaxin. Relaxin is involved in the remodeling of connective tissue and collagen and may influence the properties of ligaments, tendons, and other structures of the musculoskeletal system (34). Numerically higher plasma relaxin concentrations have been observed in women with GDM, although the difference was not statistically significant in one study, while another study reported higher relaxin-2 levels and their correlation with C-peptide concentrations in early GDM (35, 36). The possible relationship between relaxin and increased joint mobility has also been investigated in people with benign hypermobility syndrome (37). However, it is not known whether altered maternal relaxin concentrations during pregnancy can affect the development of the fetal connective and musculoskeletal systems. Therefore, the possible relationship between relaxin and the better performance in the straddle forward bend in this study should be viewed as a biological hypothesis rather than an explanation of the results. No causal relationship between maternal relaxin concentrations and offspring flexibility can be inferred from the present data. Nevertheless, this hypothesis may provide a useful starting point for future research. In addition to standardized flexibility tests, such studies could include assessments of generalized joint hypermobility, the mobility of individual joints, and connective tissue characteristics, and examine their possible association with maternal metabolic and hormonal factors during pregnancy.

The possible biological mechanisms linking prenatal exposure to GDM with later functional outcomes are multiple and still not fully understood. Glucose from the mother’s blood crosses the placenta, which may lead to fetal hyperglycemia and compensatory fetal hyperinsulinemia (2, 3). The altered intrauterine environment in GDM is not limited only to glucose. It may also include disturbances in lipid metabolism, altered adipokine and cytokine concentrations, oxidative stress, and changes in the placental transfer of nutrients (2, 3). A hyperglycemic environment may disturb the balance between oxidants and antioxidant defense mechanisms and affect cell signaling, gene expression, and inflammatory processes (2, 3). At the same time, the placenta is exposed to these metabolic changes, which may influence the transfer of glucose, amino acids, lipids, and other factors important for fetal development (2). The intensity of these effects probably depends on the timing of GDM onset, the duration and degree of hyperglycemia, the mother’s metabolic status, and the effectiveness of treatment (2, 3).

The developing brain is especially sensitive to changes in the intrauterine environment. Although a direct cause-and-effect relationship cannot be confirmed, previous studies suggest that exposure to gestational diabetes may be associated with long-term changes in brain structure and function. In children born from these pregnancies, differences in cortical thickness associated with poorer neurocognitive functioning have been reported (7), as well as selective morphological and volumetric changes in the hippocampus (8) and structural differences in the brain (9). In addition, reduced cortical excitability and neuroplasticity have been found in children aged 11 to 13 years who were prenatally exposed to GDM (10), while other studies have shown microstructural differences in the white matter of infants born to mothers with GDM (11). These changes may potentially affect the development of neural networks involved in planning, regulating, and performing movement, as well as in integrating visual and sensory information. More complex tasks, such as rolling a ball with the nondominant hand, may place greater demands on these interconnected processes and may therefore be more likely to reveal subtle functional differences. However, this study did not include brain imaging, neurophysiological measurements, or a separate assessment of executive functions. These mechanisms therefore cannot be directly linked to the motor results obtained and should instead be considered hypotheses for future multimodal longitudinal studies.

The fact that no differences were found between the groups in most motor abilities is an encouraging finding and does not support the assumption of a general motor impairment in children born from pregnancies complicated by GDM later in childhood. Although no overall group differences were observed in the balance parameters, significant group × sex interactions were identified for selected balance measures, suggesting that sex-specific patterns may warrant further exploration. However, the differences found in individual tasks suggest that more detailed monitoring of motor functioning may be useful in children born from pregnancies complicated by GDM who also show coordination difficulties, reduced motor competence, or lower participation in physical activities. The assessment should include different motor abilities and more complex functional tasks, rather than relying only on the total score of a single test. Children who are found to have difficulties should be provided with regular, developmentally appropriate, and structured activities focusing on coordination, balance, movement control, and muscular endurance. Future studies should determine whether there are specific subgroups of children born from pregnancies complicated by GDM who could particularly benefit from targeted motor programs.

## 5 Study limitation

Several limitations should be considered when interpreting these findings. First, the sex distribution differed between the groups. This imbalance reflects the composition of the original cohort available at the 10-year follow-up rather than sex-based participant selection. Sex was explicitly accounted for in the factorial ART analysis; however, the unequal group composition and relatively small subgroup sizes should still be considered when interpreting sex-related and interaction effects. The relatively small overall sample size may also have limited the statistical power to detect subtle between-group differences, and non-significant findings should therefore be interpreted with caution.

Furthermore, the GDM-exposed group included only pregnancies managed with dietary treatment, and the findings may therefore not be generalizable to offspring exposed to more severe or insulin-treated GDM. Another limitation is the long interval between the fetal assessment and the 10-year follow-up without intermediate developmental assessments. Consequently, individual developmental trajectories could not be reconstructed, and the present findings cannot distinguish between the absence of persistent effects associated with prenatal GDM exposure and possible developmental changes occurring during childhood through maturation, neuroplasticity, or environmental influences. Detailed information on children’s physical activity was collected at the 10-year follow-up; however, these data were not included in the present analyses because the relationship between physical activity patterns and motor functioning represents a distinct research question beyond the primary aim of this study. Other potentially relevant confounding factors, including socioeconomic status, maternal pre-pregnancy BMI, gestational weight gain, and maternal educational level, were not included in the present analyses; therefore, their potential contribution to the observed motor outcomes cannot be excluded. Similarly, although the cohort was established during the fetal period, the present study did not examine direct associations between individual fetal neurobehavioral measures and gross motor outcomes at the 10-year follow-up. Such associations represent a separate longitudinal research question and should be addressed in future analyses. Finally, neuroimaging, neurophysiological measures, and specific assessments of executive and visuomotor functioning were not available; therefore, the proposed biological mechanisms remain hypothetical.

## 6 Conclusion

The present 10-year follow-up study showed that gross motor functioning at approximately ten years of age was largely comparable between children prenatally exposed to gestational diabetes mellitus and children born following uncomplicated pregnancies. Nevertheless, significant group effects observed in squats, ball rolling with the non-dominant hand, and the straddle forward bend remained after accounting for sex and correction for multiple comparisons, suggesting that prenatal exposure to GDM may be associated with subtle, task-specific differences in selected domains of motor functioning during school age. These findings do not indicate a generalized impairment of gross motor development but highlight the importance of examining long-term functional outcomes using multidomain assessments that are sensitive to coordination, visuomotor integration, dynamic muscular endurance, and flexibility. Long-term developmental monitoring may be particularly relevant for children who present with additional metabolic or perinatal risk factors or emerging difficulties in motor competence and participation in physical activity. Further longitudinal studies with larger samples, more balanced group characteristics, and detailed information on maternal metabolic factors, physical activity, and neurodevelopmental functioning are needed to determine the clinical relevance and developmental stability of these findings.

## Data Availability

The raw data supporting the conclusions of this article will be made available by the authors, without undue reservation.
